# Single Origin Coffee Aroma: From Optimized Flavor Protocols and Coffee Customization to Instrumental Volatile Characterization and Chemometrics

**DOI:** 10.3390/molecules26154609

**Published:** 2021-07-29

**Authors:** Panagiota Zakidou, Fotini Plati, Anthia Matsakidou, Evdoxia-Maria Varka, Georgios Blekas, Adamantini Paraskevopoulou

**Affiliations:** 1 Laboratory of Food Chemistry and Technology, School of Chemistry, Aristotle University of Thessaloniki, 54124 Thessaloniki, Greece; zakidoup@gmail.com (P.Z.); fcplati@chem.auth.gr (F.P.); matsakidou@chem.auth.gr (A.M.); gblekas@chem.auth.gr (G.B.); 2 AVEK S.A., Naupliou 10–14, Metamorphosi, 14452 Athens, Greece; evarka@avek.gr

**Keywords:** flavor, Maillard reaction, coffee cupping, PCA, HCA, Heatmap, HS-SPME, GC-MS, geographical origin, chemometrics

## Abstract

In this study, the aroma profile of 10 single origin Arabica coffees originating from eight different growing locations, from Central America to Indonesia, was analyzed using Headspace SPME-GC-MS as the analytical method. Their roasting was performed under temperature–time conditions, customized for each sample to reach specific sensory brew characteristics in an attempt to underline the customization of roast profiles and implementation of separate roastings followed by subsequent blending as a means to tailor cup quality. A total of 138 volatile compounds were identified in all coffee samples, mainly furan (~24–41%) and pyrazine (~25–39%) derivatives, many of which are recognized as coffee key odorants, while the main formation mechanism was the Maillard reaction. Volatile compounds’ composition data were also chemometrically processed using the HCA Heatmap, PCA and HCA aiming to explore if they meet the expected aroma quality attributes and if they can be an indicator of coffee origin. The desired brew characteristics of the samples were satisfactorily captured from the volatile compounds formed, contributing to the aroma potential of each sample. Furthermore, the volatile compounds presented a strong variation with the applied roasting conditions, meaning lighter roasted samples were efficiently differentiated from darker roasted samples, while roasting degree exceeded the geographical origin of the coffee. The coffee samples were distinguished into two groups, with the first two PCs accounting for 73.66% of the total variation, attributed mainly to the presence of higher quantities of furans and pyrazines, as well as to other chemical classes (e.g., dihydrofuranone and phenol derivatives), while HCA confirmed the above results rendering roasting conditions as the underlying criterion for differentiation.

## 1. Introduction

Coffee is the second most favored beverage in the world after tea owing to its unique sensory perception by billions of consumers. Its consumption rate has been steadily increasing throughout the years, placing it among the most traded products with great economic importance on the global market. According to the International Coffee Organization (ICO), world coffee consumption for the year 2020/21 is expected to present a 1.9% increase reaching 167.58 million (60 kg) bags [1]. At the same time, the demand for high-quality coffee also presents a considerable rise with consumers, especially in Europe, being more selective in their choices, appreciating products with label of origin and searching for specific sensory attributes. The flavor of coffee is deemed the top criterion of its quality, affected by factors related to the variety, agroecological zone of its cultivation (e.g., climate, altitude, soil), as well as harvest and post-harvest processing conditions, roasting, grinding, and brewing technique [2,3]. Apart from its unique flavor, coffee offers desirable components, such as phenolic compounds, vitamins, and minerals [4].

The great significance of geographical origin and way of roasting for coffee flavor quality has been reported by many scientists [5,6,7,8]. Nowadays, consumers are increasingly attracted to coffees (blended or not) coming from from one than multiple cultivating areas, as they offer specific flavor profiles and fine coffee cup quality. The coffee industry, on the other hand, seeks ways to ameliorate the manufacturing process to meet this challenge, satisfy consumer preferences and confront growing competition [2,9,10]. In this regard, the customization of roast profiles to suit the specific characteristics of green coffee beans from certain regions or countries has enabled the achievement of the best sensory outcome, thus paving the way for the booming single-origin coffee market. Additionally, in order for the flavor uniqueness and distinctiveness of coffee to be highlighted and the final cup quality to be shaped in favor of the desired flavor quality, the approach of performing roasting separately for green coffees of different origin followed by blending is constantly gaining ground [10].

A great number of chemical compounds are likely to be involved in the flavor of roasted coffee, including volatile and non-volatile (e.g., caffeine, trigonelline, chlorogenic acids, lipids, proteins, carbohydrates, melanoidins and minerals) ones [2,11]. Although volatile compounds reach approximately 1 g/kg, they have a strong influence on coffee aroma [8,12]. Up to now, more than 1000 volatiles of several chemical classes (i.e., heterocyclic, isocyclic, and aliphatic compounds) have been identified, but, many researchers have suggested, only a small number of them (~5%) are perceptible by the olfactory receptors and play an essential role to coffee’s rich aroma [2,6,12,13]. Among these compounds, pyrazines (mostly ethyl- and ethenylalkyl-pyrazines) and furans stand out, followed by carbonyl compounds (acetaldehyde, propanal, methylpropanal, 2- and 3-methylbutanal, phenyl acetaldehyde, 2,3-butane- and 2,3-pentanedione, (Ε)-*β*-damascenone)), sulfur-containing compounds (thiols, sulfides, thiophenes), and phenol derivatives (*p*-anisaldehyde, guaiacol, 4-ethylguaiacol, 4-vinylguaiacol, vanillin), many of which are identified as coffee key odorants [3,14,15,16,17].

The majority of the volatile compounds contributing to the aroma of roasted coffee are products of Maillard reactions, including Strecker degradation of *α*-amino acids and relevant reactions, caramelization reactions, reactions occurring during roasting and resulting in the thermal degradation of carotenoids, chlorogenic and other phenolic acids, trigonelline, ascorbic acid and quinic acid [11]. On the basis of their odor quality, they can be classified in nine key odorant groups, namely (i) roasted, (ii) spices, (iii) nutty/cocoa, (iv) sweet, (v) floral, (vi) fruity, (vii) sour/fermented, (viii) green/vegetative, and (ix) other (including chemical and papery/musty odors). These are represented on the Specialty Coffee Association (SCA)’s Coffee Taster’s Flavor Wheel [18] constructed on the grounds of the World Coffee Research Sensory Lexicon [19].

Headspace solid-phase microextraction (HS-SPME) in combination with gas chromatography–mass spectrometry (GC-MS) has been successfully employed to keep track of coffee aroma contributors [3,8,20]. Additionally, acquired data can be easily chemometrically processed via mathematical and statistical tools (e.g., principal component analysis (PCA), hierarchical cluster analysis (HCA), etc.) in order to assess the underlying relationships between volatiles composition and other factors, such as geographical origin, sensory assessment attributes and processing effects [7,21,22].

In view of the above, the headspace of 10 Arabica coffee samples originating from eight different growing locations, from Central America to Indonesia, was analyzed using SPME-GC-MS as the analytical method. The samples belonged to *Coffea arabica* species and their roasting was performed under optimized temperature—time conditions, to obtain the finest, desirable sensorial characteristics of the respective brews, as these were defined by using the standard “coffee cupping” test protocol of SCA. Volatile compounds composition data were then elaborated through the HCA Heatmap, PCA and HCA aiming to depict their variation within the samples and explore if the obtained aroma profiles met the expected quality attributes, as well as if they could be an indicator of coffee origin and roasting degree.

## 2. Results and Discussion

### 2.1. Volatile Compounds Profile of the Studied Coffee Samples

**Figure 1:**
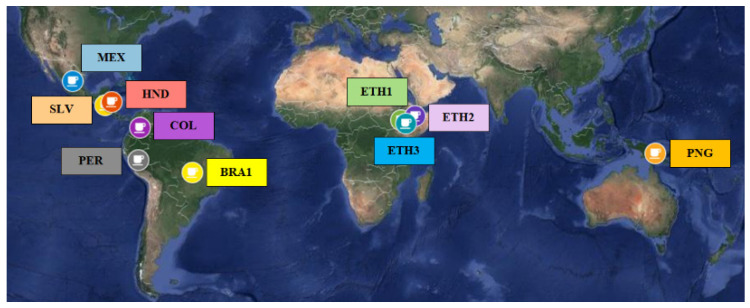
World map of single-origin coffee samples used in the study.

The volatile profile of roasted coffee samples from a wide range of geographical locations (Table 1, Figure 1) was evaluated. Headspace-SPME and GC–MS were applied, which enabled the detection of more than 130 compounds belonging to a great range of chemical classes. Specifically, a total of 138 compounds, including heterocyclic (lactones, pyranone, furan, pyrazine, pyridine, pyrrole, and thiophene derivatives), isocyclic (alcohols, aldehydes, ketones, and phenols) and aliphatic volatile compounds (aldehydes, alcohols, monocarboxylic acids and their esters, thiols, and ketones) were tentatively identified in the headspace of the samples. The volatile compounds of the analyzed samples were divided into 17 chemical groups and, along with compounds’ categorization (e.g., aliphatic aldehydes, *α*- and *γ*-diketone, norisoprenoid ketones, isocyclic aldehydes, carbonic acid, etc.), odor description and classification according to the Coffee Taster’s Flavor Wheel odorant attributes, are presented in Table 2.

Most of the volatile compounds that contribute to the aroma of roasted coffee are formed during roasting, while some of them come from the raw material (green coffee beans) without, however, participating actively in the aroma, because they are found in concentrations below their odor threshold [12]. The volatiles’ composition of the individual samples tested showed both qualitative and quantitative differences. These can be attributed to the origin of the raw material, but mainly to the roast profile at which it took place (not provided due to a non-disclosure agreement). Roasting time and temperature not only result to the intended roasting degree and color, but also cause the occurrence of chemical reactions in green coffee beans. The volatile compounds formed during roasting are products of the Maillard reaction, Strecker degradation of amino acids and related reactions, caramelization reactions, thermal decomposition of food components, such as unsaturated fatty acids, carotenoids, amino acids, hexoses, pentoses and pentose polymers, trigonelline, phenolic acids, and L-ascorbic acid, and thermal oxidative degradation of fatty acids [11]. From the detected compounds, around 30–40% was formed by the Maillard reaction, 12–16% by the Maillard reaction and thermal decomposition of food components, 16–18% by the Strecker degradation of amino acids, and 30–37% by other formation mechanisms.

The volatile compound occupying the largest percentage of the total volatiles was 5-methylfurfural (10–25%), followed by furfural and 2-furanmethanol. All of them are furan derivatives, the major class of volatile compounds present in roasted coffee samples (~25–41%). Furan derivatives are mostly formed during the Maillard reaction, but also can be products of other paths, such as thermal oxidative degradation of polyunsaturated fatty acids, degradation of thiamine and breakdown of nucleosides, dehydration of sugars during sugar caramelization, thermal degradation of carbohydrates, ascorbic acid, or unsaturated fatty acids during roasting [7,12]. 5-Methylfurfural is characterized by a caramel, maple, spicy aroma and furfural, by a sweet, woody, almond-like aroma. 2-Furanmethanol is also present in the volatile fraction of green coffee and is correlated with the undesirable burnt bitter note of dark roasted coffees, offering sweet, caramel, coffee-like notes [12]. All of them are classified in the sweet category.

Along with furan derivatives, pyrazine derivatives represent the second main class of volatile compounds contributing to the characteristic aroma of coffee (~25–39%). These compounds are mainly derived by Strecker degradation of *α*-amino acids. Twenty-two pyrazines were found in the coffee samples, among which 2-ethyl-3,5-dimethylpyrazine, 3-ethyl-2,5-dimethylpyrazine, methylpyrazine, 2,5-dimethylpyrazine and 2,6-dimethylpyrazine. They are all considered as potent odorants in coffee, characterized by nutty, cocoa, roasted odor notes, and consequently, classified in the nutty/cocoa category. The coffee samples from Honduras (HND) and Brazil (BRA1) were found to contain the highest levels of pyrazine derivatives with 36.1% and 39.1%, respectively, that is captured by the resulting nutty scent of their brews (Table 2).

Esters were found to represent the third most abundant class of volatile compounds identified in the volatile fraction of the examined roasted coffee samples (5–8%), mainly due to the presence of 2-furanmethanol acetate (3.7–5.8%) followed by 2-furanmethanol propanoate (0–1.3%), conferring the fruity aroma of the roasted samples.

Pyrrole derivatives (~5–12%) are a minor class of volatile compounds present in roasted coffee. It seems that they are formed by reactions of aldoses with alkylamines and may result from the thermal degradation of *Amadori* intermediates, while caramelization, pyrolysis and trigonelline’s degradation may also take place [9,12]. The most abundant in the volatile fraction of the examined roasted coffee samples was 1-methyl-1H-pyrrole-2-carboxaldehyde, followed by 2-acetylpyrrole and 2-formylpyrrole, three Maillard reaction products with roasted, nutty notes, contributing to the nutty/cocoa category.

There is little information available regarding the contribution of pyridine derivatives (~3–6%) to the roasted coffee aroma. They can be produced by thermal degradation of *Amadori* intermediates, but also by pyrolysis of amino acids and trigonelline [12]. The compound with the highest percentage was pyridine itself, a trigonelline degradation product, with fishy, sour notes, part of the sour/fermented category.

Aldehydes and ketones have been found to represent ~1–6% of the volatile organic compounds present in different roasted coffee samples; however, single compounds do not exceed more that 1% of the total sample contribution. Aldehydes of different chain length present different sensory characteristics and can be formed by the Maillard reaction, by the oxidative degradation of amino acids during their interaction with sugars at high temperatures (Strecker degradation), and during the interaction of amino acids and polyphenols in the presence of polyphenol oxidase at normal temperatures [12]. The main pathway for ketones formation in the raw coffee seeds is the oxidation of fatty acids [26]. They are formed during roasting as Maillard reaction and caramelization products. The formation of volatile aldehydes and ketones has also been attributed to self-oxidation of alcohols and autoxidation of unsaturated fatty acids via the breakdown of hyperoxide intermediates [7,12].

Phenol derivatives (~2–4%) are important contributors to the aroma of roasted coffee. They are formed by the thermal degradation of chlorogenic acids, such as ferulic, caffeic and quinic acids, lignin and decarboxylation of phenolic carboxylic acids during roasting [12]. Phenol, 2-methoxy-phenol (guaiacol), 2-methoxy-4-ethyl-phenol (4-ethylguaiacol), and 2-methoxy-4-vinylphenol (4-vinylguaiacol), with spicy or phenolic aroma, and vanillin, with sweet or vanilla-like notes, were found to be the main phenolic compounds present in the roasted coffee samples studied.

Sulfur-containing compounds, such as thiols, sulfides and thiophenes, are among the key odorants of coffee aroma, regardless of their quite low concentration [2]. 2-Furfurylthiol, described as having a strong, fresh roasted coffee aroma, has been reported to be of great importance because of its very low sensory threshold. The coffee samples COL, HND and PER were characterized by the highest percentage of 2-furfurylthiol (~0.3%) compared to all the other samples, which denotes that the roasted-like odor will affect more intensely their aroma (Table 1) [15]. Other S-containing compounds, such as furfuryl methyl sulfide and furfuryl methyl disulfide, were also detected in the headspace of the samples exhibiting alliaceous/vegetable and roasted characters, respectively [14,27].

Carbonic acids (~1.5–4%), are not crucial to coffee aroma. Their contribution could be either positive with cheese, cream, chocolate notes or negative with sweat-like notes [12]. The most abundant was 3-methyl-2-butenoic acid with phenolic, dairy, green odor notes.

Other volatile compounds identified in all roasted coffee samples were maltol (1.7–3.8%), a pyranone derivative with sweet, caramel-like, cotton candy notes, and 2,5-dimethyl-4-hydroxy-3(2H)-furanone (furaneol) (0.3–0.7%), a dihydrofuranone derivative with caramel-like, cotton candy notes, which are Maillard reaction products, and *γ*-butyrolactone (0.1–0.3%) related to creamy, fatty, caramel odor were found in all samples. Monoterpenes, such as *β*-myrcene, *d*-limonene and linalool (associated with spicy, citrus and floral notes, respectively), were identified in almost all samples, but probably without any significant impact on their aroma due to their rather high odor thresholds [28].

Concerning the contribution to the coffee aroma, some of the identified compounds present an extremely low odor threshold in air, such as (E)-*β*-damascenone (2–4 pg/L), 2-ethyl-3,5-dimethylpyrazine (7–14 pg/L), 4-ethyl-guaiacol (10–30 pg/L) and 2-furfurylthiol (10–20 pg/L) or high odor threshold in air, such as 4-vinylguaiacol (0.4–0.8 ng/L), vanillin (0.6–1.2 ng/L), 2,5-dimethyl-4-hydroxy-3(2H)-furanone (0.5–1.5 ng/L), 3-methyl-butanal (2–4 ng/L), 3-ethyl-2,5-dimethylpyrazine (2.4–4.8 ng/L), 2,3-butanedione (10–20 ng/L) and 2,3-pentanedione (10–20 ng/L) [28]. Other compounds, such as Strecker degradation aldehydes of branched amino acids (isoleucine, and leucine), namely 2-methylbutanal and 3-methylbutanal, 2,3-pentanedione, 2-methoxyphenol (guaiacol) and pyrazine derivatives (ethylpyrazine, 2-ethyl-6-methylpyrazine and 2-ethyl-3,5-dimethylpyrazine) have also been reported as key odorants in literature in espresso beverages and coffee silver skin [3,20].

### 2.2. Volatile Odor Description and Categorization—Heatmap Analysis

The sample with the greatest number of volatile compounds (114) was COL, probably due to its dark roast. Other medium-roasted coffee samples, such as HND, PER, ETH2, also contained a great amount of volatile compounds (111, 111, 102, accordingly), while 80–94 volatile compounds were detected in the headspace of the rest samples (ETH1, ETH3, BRA1, SLV, MEX, PNG).

Volatile compounds were characterized using odor descriptors extracted from the Good Scents Company Information System [23], Flavornet [24] and The Pherobase [25] online databases. Moreover, all volatile compounds detected in the coffee samples were categorized according to the SCA Coffee Taster’s Flavor Wheel [18], into nine categories—specifically, roasted, spices, nutty/cocoa, sweet, floral, fruity, sour/fermented, green/vegetative and other (containing chemical and papery/musty odors)—on the basis of each compound’s odor descriptors. Co-eluted compounds of different characterization were not included.

Most of the identified compounds belong to the category “nutty/cocoa” (20–24 volatiles in every sample), which mainly consists of pyrazine, furan, and pyrrole derivatives, followed by the “sweet” category (10–18 volatiles in every sample) delivered by aldehydes and ketones and other (6–16 volatiles in every sample). The contribution of the “other” category is endorsed by different compounds, such as furan, thiophene, and pyrrole derivatives, aldehydes, ketones, esters, and thiols. The “floral” category consists mostly of alcohols, ketones, monoterpenes, pyrrole derivatives, “fruity” category of aldehydes, ketones, and esters, “green/vegetative” category of pyridine, pyrazine, aldehydes and furan derivatives, “roasted” category of pyrazine, pyridine, furan, and thiophene derivatives, “sour/fermented” category of acids, and “spices” category of furan derivatives.

A Hierarchical Cluster Analysis Heatmap (HCA Heatmap) was constructed to illustrate the similarities and differences among the studied roasted coffee samples in terms of the volatile compound odor categories and to observe a correlation with desired brew characteristics. The dataset consisted of 10 observations (roasted coffee samples) and nine variables (volatile compound categories).

**Figure 2:**
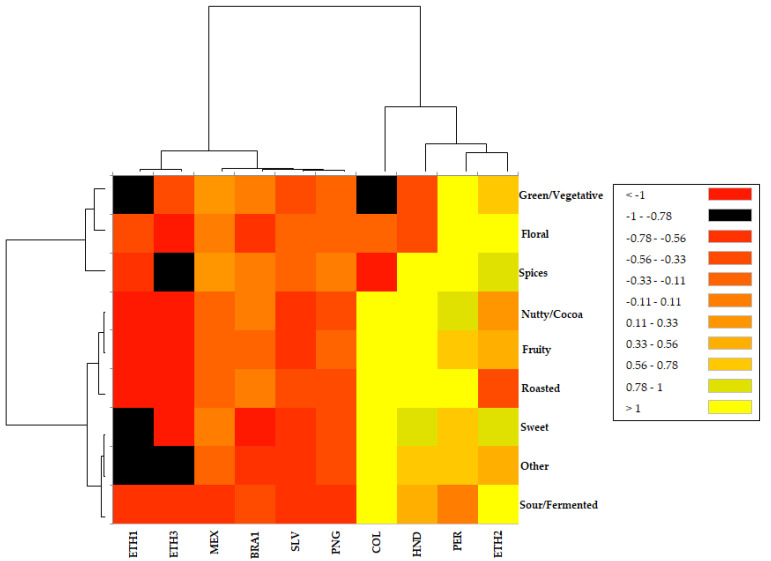
Hierarchical Cluster Analysis Heatmap (HCA Heatmap) of the volatile categories characterizing roasted coffee samples. The color of each tile of the Heatmap indicates the type/strength of the correlation for a given aroma category/sample combination. The yellow color indicates major abundance, while the red color indicates minor abundance.

As Figure 2 reveals, the nine volatile compound categories were clearly divided into two clusters. Cluster I contained spices, floral and green/vegetative categories, with an inner subcategory differentiating spices from the other two categories. Cluster II contained nutty/cocoa, fruity, roasted, sweet, other and sour/fermented categories. Subcategories divided nutty/cocoa, fruity and roasted from the rest of the categories sweet, other and sour/fermented. The main clustering is affected by the percentage of volatiles in each flavor category and cannot be connected to the chemical classes included in the categories. The percentage of compounds in Cluster I is much lower compared to Cluster II.

It can be observed that aroma contribution is higher in dark- and medium-roasted samples COL, HND, PER, ETH2. COL, although the sample with the darkest roast, seems to be inferior to the categories of the Cluster I, mostly spices, green/vegetative and, to a lesser extent, floral, while the rest are in high abundance. COL is also high in nutty/cocoa and roasted-smelling compounds, which is consistent with its desired brew characteristics of nutty and toasted notes (Table 1). ETH1 and ETH3 are the lightest roasted samples, with a minor abundance of volatiles, specifically of those included to nutty/cocoa, fruity and roasted categories. As described in the brew characteristics of ETH1, “floral” seems to be the category with the highest contribution; however, an estimation cannot be made for ETH3. In MEX, BRA, SLV and PNG, the orange tiles of medium abundance seem to excel. The HND, PER and ETH2 samples are described by major abundance yellow and yellowish tiles. HND is mostly characterized by spices, nutty/cocoa, fruity, roasted, but also sweet odors, in accordance to its vanilla, butter, caramel, nuts notes and chocolate taste and PER by green/vegetative, floral, spices and roasted-smelling volatiles, in accordance to its spicy, floral, citrus aroma. The aroma profile of ETH2 is comprised mostly by floral and sour/fermented volatile compounds, but also by fruity to a great extent, in agreement with its berries, rose, floral, or apricot notes. In general, a decent correlation could be observed between samples and their brew characteristics.

### 2.3. Sample Differentiation—Principal Component Analysis (PCA) and Hierarchical Cluster Analysis (HCA)

**Figure 3:**
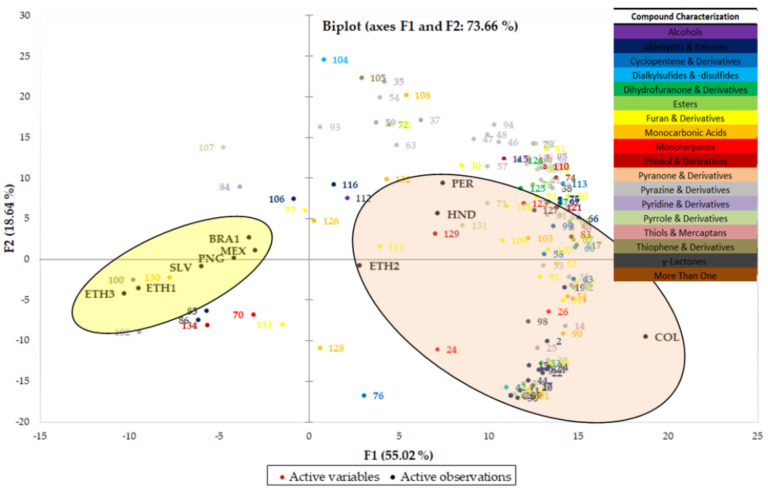
Principal component analysis (PCA) scores plot for coffee samples (active observations, black) based on volatile compounds (active variables, multicolored).

Unsupervised PCA clustering method was utilized to map the natural groupings of coffee samples based on the abundances of the detected volatile compounds. The distribution of the volatile compounds within each coffee sample, as well as the relationships between them, is reflected in the associated PCA score plot, shown in Figure 3. For better monitoring, all volatile compounds were numbered, grouped into chemical categories, and colored according to the enclosed table.

Overall variance of the first two principal components is 73.66%, of which 55.02% relates to F1 and 18.64% to F2. Clustering conformation of roasted coffees is dominated by roasting degree as the main discrimination parameter. In particular, PCA score-plot clustered coffee samples in two main subgroups. The first component (F1) with positive loading (right area) separated the first subgroup (highlighted with a pink-colored circle) of dark and medium roasted samples (COL, ETH2, HND, PER), with respect to the second group (highlighted with a yellow-colored circle) that includes mostly light and medium roasted samples (ETH1, ETH3, SLV, MEX, PNG, BRA1) with a negative loading on F1 (left area). In the first subgroup on the right area of the score plot (F1 with positive scoring), F2 plotted medium roasted samples PER and HND samples in the upper quadrant with positive loading, while the medium/dark ETH2 and dark COL were located in the lower quadrant with negative loading, with COL being clearly distant from the other samples. In the second subgroup on the left side of the score plot (F1 with negative scoring), F2 plotted medium roasted BRA1 and medium/light roasted MEX and PNG samples in the upper quadrant with positive loading, while the medium light roasted SLV and light roasted ETH1 and ETH3 were in the lower quadrant with negative loading. The medium/light roasted SLV sample was noticed to be closer to medium/light roasted PNG, than ETH1 and ETH2, the lightest roasted samples.

As can be observed by the score plot, darker roasted samples (pink-colored circle) were influenced heavily by compounds belonging to the furan and pyrazine derivatives (yellow and light grey colored active observations, respectively), dihydrofuranone and phenol derivatives (green and deep red colored symbols) as well as *γ*-lactones (deep grey colored symbols), while they were also related to more volatile compounds than lighter roasted samples. These compound categories are associated with dark roasting and sweet, nutty, cocoa, caramel, spicy notes, observed mostly in COL and HND brew characteristics (Table 1).

Volatile compounds, considered as coffee key odorants, were selected to be discussed. The negative portion of the F1 axis (left area) was related to (E)-*β*-damascenone (106) and vanillin (134) with a high contribution to the aroma of roasted coffee, characterizing mostly light roasted samples, which have floral, sweet, fruity and sweet, vanilla odor descriptors, respectively. The positive portion of the F1 axis (right area) was related mainly to 2,5-dimethyl-4-hydroxy-3(2H)-furanone (124, caramel, sweet, cotton candy), 4-ethylguaiacol (123, spicy, phenolic), 4-vinylguaiacol (129, spicy, clove, phenolic) (positive portion of the F2 axis, upper area) and 3-ethyl-2,5-dimethylpyrazine (53, nutty, cocoa, roasted), 3-methyl-butanal (9, aldehydic, fruity, green), 2,3-pentanedione (17, sweet, caramel, buttery), 2,3-butanedione (11, sweet, caramel, buttery) (negative portion of the F2 axis, lower area), all important roasted coffee aroma markers. The latter clearly defined dark or medium roasted samples COL, HND and ETH2 samples.

Although PCA showed a natural separation of coffee samples by roasting degree and a correlation to desired brew characteristics, it did not demonstrate a separation between samples based on their geographical origin [5].

**Figure 4:**
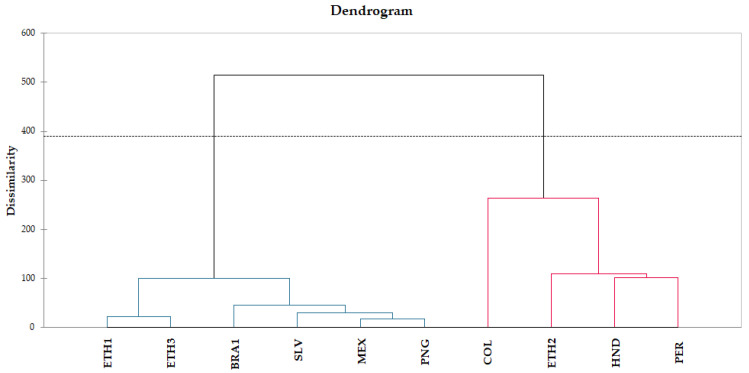
Hierarchical cluster analysis (HCA) of coffee samples.

Similarly, hierarchical cluster analysis (HCA) was applied to the set of variables employed for PCA, in order to interpret the results in a graphical way. *X*-axis represents the analyzed coffee samples and *y*-axis shows the dissimilarity (Figure 4). Clustering of roasted coffee samples presented a grouping by roasting degree and expected brew characteristics and, secondarily, geographical origin. Specifically, a clear clustering of two distinct groups was observed among coffee samples of different roasting degree in accordance with PCA results. The first blue cluster contains samples with lighter roast (ETH1, ETH3, BRA1, SLV, MEX, PNG) and the second red cluster, samples with darker roast and more intense roasting conditions (COL, ETH2, HND, PER). Within each group, the effect of geographical origin can be partially observed.

In the first group, samples are clustered from Africa with light roast (ETH1, ETH3) and from America and Australia with medium/light or medium roast. In a second clustering, South American, medium roasted sample BRA1 is differentiated from medium/light roasted North American samples MEX, SLV and Australian PNG.

In the second group containing the darker roasted coffee samples, geographical origin is a secondary criterion for differentiation. South American COL is clustered alone due to its intense dark roast and volatile compounds’ abundance, compared to African medium/dark roasted ETH2 and, in the next grouping, medium roasted North American HND and South American PER.

The similarity between samples inside each major group indicates that roasting degree exceeds the geographical origin of coffee, meaning that volatile composition is affected by roasting conditions to a greater extent than by origin and coffee bean composition. For example, coffee samples from Ethiopia (ETH1 and ET3) belong to the same inner group, while ETH2 can be observed to the second district group, due to its more intense roast. It then became apparent that PCA and HCA multivariate statistical analyses were able to adequately differentiate samples according to their roasting degree, considering their volatile composition.

## 3. Materials and Methods

### 3.1. Coffee Samples

Ten Arabica (*Coffea arabica* L.) coffee samples were provided by AVEK S.A. (Athens, Greece). The samples were of single origin, covering a wide geographical distribution from Central America to Indonesia (i.e., Brazil, Colombia, El Salvador, Ethiopia, Honduras, Mexico, Papua New Guinea, Peru) (Figure 1). Table 1 lists the production country and region, roasting degree, and brew sensory characteristics of the studied samples.

Roasting was accomplished by the company using a custom-made fluidized bed roaster (Coffeetool, Athens, Greece). Charge temperature for all samples was 190 °C following a 14 min reference profile with the end temperature being between 200–220 °C. The roasting conditions applied were the result of preliminary roasting trials, were unique for each coffee sample, and were aimed to reach the desirable cup quality shown in Table 1. Their selection was based on the sensory evaluation of the respective espresso coffee brews conducted according to SCA Cupping protocols [29]. A typical time–temperature roasting curve is available as Supplementary Material (Figure S1). Due to confidentiality reasons, detailed roasting conditions were not disclosed by the company.

Roasted coffee samples were immediately air-cooled and allowed to rest for 1 h, before grinding with a Swiss Ditting grinder for 10 s in order to pass a ~500 μm sieve, and were stored in sealed containers under nitrogen atmosphere at 4 °C for a maximum of 24 h. Prior to HS-SPME analysis, the coffee samples were brought to room temperature (1 h).

### 3.2. Volatile Compounds Analysis

The volatile compounds of the coffee samples were analyzed by HS-SPME/GC-MS according to the method described by Lee et al. [30] and Papageorgiou et al. [31], with certain modifications. Specifically, 1.2 g of ground coffee were placed into a 15 mL vial, which was immediately sealed with a Teflon-lined septum and screw cap (Supelco, Bellefonte, PA, USA). A minimum time of 10 min was determined for equilibration, during which the samples were incubated at 50 °C under magnetic stirring. The headspace of the ground coffee was then sampled (30 min) by using an SPME fiber coated with CAR/PDMS (75 μm) (Supelco, Bellefonte, PA, USA), pre-conditioned according to manufacturer recommendations. During the extraction process the sample was continuously magnetically stirred. Afterwards, the fiber was thermally desorbed at 250 °C for 5 min in splitless mode. Carry-over effects were diminished by holding the fiber in the injection port for an extra 5 min.

Volatile compounds analysis was performed using an Agilent 6890A gas chromatograph equipped with MSD5973 mass spectrometer (Palo Alto, USA). The volatile compounds were separated on a polar DB-Wax column (60 m × 0.32 mm i.d., film thickness 0.25 μm; Agilent J&W, Palo Alto, CA, USA). Helium was used as carrier gas at a constant flow rate of 1.0 mL/min. The oven temperature was set at 50 °C for 5 min, followed by an increase of 3 °C/min up to 230 °C (15 min) (total run time 80 min). The transfer line temperature was set at 240 °C. MS was taken at 70 eV with a scan range between 35 and 500 amu at 2 scans/s. The MS source and MS Quad temperatures were 230 °C and 150 °C, respectively. Compounds identification was based on the comparison of the experimental mass spectra with those stored in NIST (Version 2.0g, 2011) and AMDIS libraries considering a similarity level >800 as well as on the comparison of calculated GC retention indices (RI) (using C7-C30 alkanes, Sigma-Aldrich, Laramie, WY, USA) with RIs reported in the literature. Data were processed by the MSD ChemStation software and expressed as relative percentage of each compound peak area to the total GC-MS peak area. The samples were evaluated in triplicate and the coefficient of variance (CV) for the vast majority of the compounds was lower than 6.5%. The suitability of the analysis conditions and the absence of contaminants were verified by running blank samples every two injections.

### 3.3. Statistical Analysis

The results were expressed as a mean of at least three measurements (*n* = 3). Multivariate statistical analysis, specifically Principal Component Analysis (PCA), Hierarchical Cluster Analysis (HCA) and Hierarchical Cluster Heatmap Analysis (HCA-Heatmap), was performed using XLSTAT 2021.2 (Addinsoft, New York, NY, USA). Pearson correlation was adopted.

## 4. Conclusions

In this work, the headspace composition of 10 single origin Arabica coffee samples from different geographical regions, roasted separately to different roasting degrees under varying conditions adapted for each sample to reach specific sensory attributes of their brews, was successfully profiled using SPME/GC–MS combined with chemometrics. Volatile compounds differed for the different roasted coffees and showed a strong variation with the applied roasting profile. A total of 138 volatile compounds were tentatively identified in all samples, of which, pyrazine and furan derivatives were predominant. The desired brew characteristics were satisfactorily captured from the volatile compounds formed, contributing to the aroma potential of each sample as chemometric analysis revealed. Furthermore, lighter roasted samples were efficiently differentiated from darker roasted samples, while roasting degree prevailed over coffee’s geographical origin. In general, the concept of customizing roast profiles to highlight the finest features of single origin coffee samples, and obtain the best aroma profile and thus sensory outcome, determines the differentiation between samples, and allows their ensuing blending and tailoring of cup quality thus providing a valuable tool for the coffee industry.

## Figures and Tables

**Table 1:** Description of the studied coffee samples.

Sample Label	Origin	Roasting Degree ^1^	Brew Characteristics
COL	Colombia	Dark	Aroma: woody, spicy, toasted notes with nutty hints Acidity: gentle brightness Flavor: butter, caramel, chocolate, nuts Body: moderate body Aftertaste: dark chocolate with nutty hints
HND	Honduras	Medium	Aroma: vanilla, butter, caramel, nutty notes Acidity: moderate brightness Flavor: butter, caramel, chocolate, spicy, toast-like notes Body: full body Aftertaste: chocolate, nutty, sherry
MEX	Mexico	Medium/Light	Aroma: spicy, woody, vanilla notes Acidity: gentle brightness Flavor: sandalwood, honey, pineapple, vanilla Body: medium body Aftertaste: dark chocolate hints, vanilla
SLV	El Salvador	Medium/Light	Aroma: spicy, fruity, popcorn, toasted notes Acidity: gentle brightness Flavor: mocha, caramel, apple, nutty, almonds Body: moderate body Aftertaste: dark chocolate hints, nutty
PER	Peru	Medium	Aroma: spicy, floral, citrus notes Acidity: gentle brightness Flavor: tobacco, caramel, vanilla, nutty Body: moderate body Aftertaste: velvety, sweet, caramel
PNG	Papua New Guinea	Medium/Light	Aroma: spicy, apricot, bouquet notes Acidity: moderate brightness Flavor: woody, spicy, rose, honey, apricot, malty Body: full body Aftertaste: dark chocolate hints, butter, caramel
BRA1	Brazil	Medium	Aroma: toasted notes with nutty hints Acidity: gentle brightness Flavor: caramel, chocolate, nutty Body: full body Aftertaste: dark chocolate
ETH1	Ethiopia, Sidamo	Light	Aroma: citrus, bouquet notes Acidity: sparkle brightness Flavor: lemon, caramel, berries Body: medium body Aftertaste: dark chocolate hints
ETH2	Ethiopia, Harrar	Medium/Dark	Aroma: berries, rose, floral, apricot notes Acidity: moderate brightness Flavor: butter, caramel, apricot, rose bouquet Body: full body Aftertaste: dark chocolate hints, caramel

**^1^** The roasting degree was evaluated with a ColorTrack Model R-100 Color Laser Analyzer (QCAC LAB LLC, Kilmarnock, VA, USA).

**Table 2:** Volatile compounds identified in the studied coffee samples by headspace solid-phase microextraction with gas chromatography–mass spectrometry (hs-spme/gc-ms).

No ^a^	Volatile Compound	RI ^b^	Compound Category ^c^	Odor Description ^d^	Coffee Taster’s Flavor Wheel Classification	Relative Content (%) ^e^									
						COL	HND	ETH1	PER	MEX	SLV	ETH2	ETH3	BRA	PNG
**Pyrazine and Derivatives**															
28	Pyrazine	1223	PZ	nutty, roasted	nutty/cocoa	0.49	0.14	-*^f^*	0.09	-	-	0.33	-	-	-
33	Methylpyrazine	1281	PZD	nutty, cocoa, roasted	nutty/cocoa	5.03	4.49	0.87	3.25	2.38	1.33	4.99	0.52	0.72	1.73
38	2,5-Dimethylpyrazine	1336	PZD	cocoa, roasted, nutty	nutty/cocoa	3.62	5.30	3.97	4.17	4.73	4.88	3.88	3.21	5.26	4.66
39	2,6-Dimethylpyrazine	1343	PZD	cocoa, roasted, nutty	nutty/cocoa	3.63	5.51	3.01	4.12	4.35	4.07	3.64	2.53	4.96	4.35
40	Ethylpyrazine	1348	PZD	nutty, roasted, cocoa	nutty/cocoa	1.69	2.37	0.96	1.58	1.63	1.26	1.61	0.74	1.48	1.56
41	2,3-Dimethylpyrazine	1360	PZD	nutty, roasted, cocoa	nutty/cocoa	0.83	1.40	0.48	0.94	0.91	0.74	0.83	0.38	1.25	0.97
46	2-Ethyl-6-methylpyrazine	1396	PZD	cocoa, roasted	nutty/cocoa	1.68	3.24	2.07	2.47	2.68	2.91	1.61	2.03	4.15	2.59
47	2-Ethyl-5-methylpyrazine	1404	PZD	coffee, nutty	nutty/cocoa	1.18	2.10	2.45	1.74	1.92	2.28	1.27	1.76	3.47	2.41
48	2-Ethyl-3-methylpyrazine	1408	PZD	nutty	nutty/cocoa	1.66	3.14	2.19	2.61	2.61	3.14	1.79	2.23	4.88	2.65
	Trimethylpyrazine			nutty, cocoa, potato, roasted											
49	Propylpyrazine	1432	PZD	green, vegetable, nutty	green/vegetative	0.18	0.22	0.12	0.18	0.14	0.17	0.14	0.13	0.30	0.14
51	Vinylpyrazine	1448	PZD	nutty	nutty/cocoa	0.66	0.93	0.95	0.88	0.88	1.05	0.70	1.03	1.37	0.85
53	3-Ethyl-2,5-dimethylpyrazine	1462	PZD	nutty, cocoa, roasted	nutty/cocoa	1.61	1.09	1.42	1.51	1.61	2.03	0.56	1.50	1.92	1.24
54	2,3-Diethylpyrazine	1468	PZD	raw, nutty	green/vegetative	-	0.08	0.08	0.08	-	-	-	-	0.09	-
56	2-Ethyl-3,5-dimethylpyrazine	1474	PZD	nutty, burnt, roasted, coffee	nutty/cocoa	0.47	0.69	0.64	0.67	0.68	0.75	0.57	0.61	0.85	0.64
57	2-Methyl-6-propyl-pyrazine	1477	PZD	burnt, nutty, roasted	nutty/cocoa	0.17	0.25	0.00	0.23	0.21	-	-	-	0.43	0.23
61	2-Methyl-6-vinylpyrazine	1499	PZD	nutty	nutty/cocoa	0.47	0.38	0.72	0.65	0.59	0.77	0.52	0.82	0.82	0.54
63	2-Methyl-5-vinylpyrazine	1502	PZD	coffee	roasted	0.39	0.64	0.93	0.82	0.75	1.06	-	1.08	1.04	0.70
74	(1-Methylethenyl)-pyrazine	1574	PZD	caramel, chocolate, nutty, roasted	sweet	0.36	0.61	0.83	0.74	0.65	0.85	0.40	0.92	0.91	0.62
83	Acetylpyrazine	1645	PZD	popcorn, nutty, coffee, roasted	nutty/cocoa	1.02	1.14	0.88	1.07	0.79	1.03	1.05	0.91	1.1	0.83
93	6-Methyl-2-acetylpyrazine	1706	PZD	roasted, coffee, cocoa, popcorn	nutty/cocoa	-	1.27	1.65	1.13	-	-	-	1.93	2.14	-
94	5-Methyl-2-acetylpyrazine	1715	PZD	nutty, popcorn	nutty/cocoa	0.90	1.14	2.21	1.69	1.56	2.29	1.22	2.51	1.98	1.66
	$\sum^{\;}\mathrm{Pyrazine}\;\mathrm{and}\;\mathrm{derivatives}$					**26.04**	**36.13**	**26.43**	**30.62**	**29.07**	**30.61**	**25.11**	**24.84**	**39.12**	**28.37**
**Furan and Derivatives**															
3	Furan	801	F	ethereal	other	0.14	-	-	-	-	-	-	-	-	-
6	2-Methylfuran	878	FD	cocoa, nutty, coffee	nutty/cocoa	1.13	0.06	-	0.06	0.04	-	-	-	-	0.04
10	2,5-Dimethylfuran	959	FD	meaty, roasted	other	0.29	0.04	-	-	-	-	0.06	-	-	-
18	Vinylfuran	1087	FD	phenolic, coffee	other	0.25	0.07	*-*	0.05	0.02	-	0.13	-	-	0.03
27	2-(2-Propenyl)furan	1219	FD	- *^f^*	- *^f^*	0.35	0.32	0.18	0.22	0.20	0.13	0.26	0.18	0.19	0.22
30	2-Pentylfuran	1245	FD	green, earthy, beany, vegetable	green/vegetative	0.05	0.16	-	0.07	0.15	-	0.05	-	-	0.10
31	2-(Methoxymethyl)-furan	1247	FD	coffee, roasted	roasted	0.24	0.07	-	0.04	-	-	0.13	-	-	-
55	Furfural	1470	FD	sweet, woody, almond	sweet	7.76	7.49	5.80	6.52	8.10	4.39	9.00	3.68	1.88	7.15
64	Acetylfuran	1510	FD	nutty, sweet, cocoa, coffee	nutty/cocoa	3.67	4.14	3.35	3.50	4.10	3.26	3.44	2.87	2.79	4.10
73	5-Methylfurfural	1573	FD	caramel, maple, spicy	sweet	10.36	12.34	24.99	14.53	18.80	19.06	12.85	23.68	14.07	19.52
74	1-(2-Furanyl)-1-propanone	1574	FD	fruity	fruity	0.33	0.24	0.24	0.23	0.33	0.50	0.33	0.32	0.14	0.45
77	2,2’-Bifuran	1590	FD	meaty, sulfury	other	-	0.39	-	-	0.45	-	-	0.72	-	-
80	2,2’-Methylenebis-furan,	1618	FD	roasted	roasted	0.96	1.08	1.21	1.22	1.15	1.41	0.83	1.42	1.45	1.21
81	2-Acetyl-5-methylfuran	1625	FD	nutty, musty	nutty/cocoa	0.42	0.54	0.78	0.67	0.64	0.74	0.50	0.90	0.77	0.70
89	2-Furanmethanol	1672	FD	caramel, coffee	sweet	8.76	6.03	1.76	4.95	3.09	1.77	9.28	1.57	1.14	2.60
91	2-Furfuryl-5-methylfuran	1682	FD	-	-	0.27	0.17	0.29	0.31	0.21	0.33	0.19	0.27	-	0.19
92	1-(5-Methyl-2-furanyl)-1-propanone	1701	FD	green, nutty	green/vegetative	-	-	-	0.41	-	-	-	-	-	-
96	3,4-Dimethyl-2,5-furandione	1757	FND	-	-	0.62	0.68	0.84	0.92	0.85	1.00	0.72	1.05	1.19	0.88
109	3-(2-Furanyl)-2-propenal (3-(2-Furyl)acrolein)	1860	FD	spicy, woody, cinnamon	spices	0.23	0.18	0.52	0.33	0.38	0.45	0.30	0.57	0.28	0.35
111	2-Methyl-3(2-furyl) acrolein (cinnamon acrolein)	1871	FD	spicy, cinnamon, woody	spices	0.08	0.06	0.14	0.12	0.10	0.23	-	0.25	0.10	0.11
114	4-(2-Furanyl)-3-buten-2-one	1907	FD	spicy, cinnamon	spices	0.10	0.09	0.21	0.17	0.14	0.21	0.13	0.26	0.14	0.16
120	Furan, 2,2’-[oxybis(methylene)]bis- (Furfuryl ether)	1985	FD	coffee, nutty, earthy	nutty/cocoa	0.12	0.08	0.16	0.16	0.12	0.19	0.14	0.19	0.13	0.13
133	5-Hydroxymethylfurfural	2517	FD	buttery, caramel	sweet	0.04	0.03	0.19	0.06	0.08	0.11	0.06	0.15	0.01	0.08
	$\sum^{\;}\mathrm{Furan}\;\mathrm{and}\;\mathrm{derivatives}$					**36.17**	**34.26**	**40.66**	**34.54**	**38.95**	**33.78**	**38.4**	**38** **.0** **8**	**24.28**	**38.02**
**Esters**															
5	Methyl acetate	833	ES	fruity, fresh, sweet	fruity	0.20	0.02	-	0.02	-	-	-	-	-	-
62	Furfuryl formate	1500	ES	ethereal	other	1.16	0.96	0.43	0.73	0.66	0.53	1.09	0.37	0.46	0.71
67	1-(Acetyloxy)-2-butanone	1538	ES	-	-	0.76	0.87	0.48	0.66	0.63	0.57	0.73	0.50	0.66	0.77
68	2-Furanmethanol, acetate	1542	ES	fruity, sweet	fruity	4.44	5.14	3.71	4.44	4.63	4.75	4.75	3.77	5.58	5.76
72	2-Furancarbothioic acid, S-methyl ester	1562	ES	alliaceous, onion, cabbage	green/vegetative	-	-	-	0.05	-	-	-	-	-	-
78	2-Furanmethanol propanoate	1605	ES	fruity, sweet	fruity	0.74	0.84	-	0.98	0.93	1.29	0.77	1.26	1.34	1.05
	$\sum^{\;}\mathrm{Esters}$					**7.30**	**7.83**	**4.62**	**6.88**	**6.85**	**7.14**	**7.34**	**5.90**	**8.04**	**8.29**
**Pyrrole and Derivatives**															
23	1-Methyl-1H-pyrrole	1149	PRD	woody, herbal, smoky	other	0.32	0.02	-	-	-	-	0.15	-	-	-
65	Pyrrole	1520	PR	nutty, sweet	nutty/cocoa	0.15	-	-	-	-	-	0.10	-	-	-
71	2-Methylpyrrole	1560	PRD	-	-	0.14	0.18	-	0.20	-	-	0.14	0.33	0.38	-
82	1-Methyl-1H-pyrrole-2-carboxaldehyde	1641	PRD	roasted, nutty	nutty/cocoa	1.68	2.24	3.33	2.59	2.88	3.14	2.26	3.46	3.61	2.98
88	2-Acetyl-1-methylpyrrole	1670	PRD	earthy	other	0.67	0.77	1.00	0.95	0.94	1.26	0.72	1.23	1.47	1.00
107	1-(Furan-2-ylmethyl)pyrrole (1-Furfurylpyrrole)	1834	PRD	vegetable, green	green/vegetative	-	-	2.27	2.07	2.09	2.16	1.61	2.45	2.08	2.05
119	1-(1H-Pyrrol-2-yl)ethenone (2-Acetylpyrrole)	1975	PRD	musty, nutty	nutty/cocoa	1.21	1.28	2.27	1.96	1.90	2.46	1.53	2.68	2.50	1.81
122	1H-Pyrrole-2-carboxaldehyde (2-Formylpyrrole)	2031	PRD	musty, coffee	nutty/cocoa	0.90	1.01	2.10	1.52	1.65	1.62	1.19	2.14	1.45	1.48
131	Indole	2468	PRD	floral	floral	0.02	-	-	0.04	0.03	-	0.03	-	-	0.04
	$\sum^{\;}\mathrm{Pyrrole}\;\mathrm{and}\;\mathrm{derivatives}$					**5.09**	**5.5** **0**	**10.97**	**9.33**	**9.49**	**10.64**	**7.73**	**12.29**	**11.49**	**9.36**
**Pyridine and Derivatives**															
14	1,2,3,6-Tetrahydro-1-methyl-pyridine	1036	PDD	caramel	sweet	0.09	0.04	-	0.04	-	-	0.06	-	-	-
25	Pyridine	1196	PD	fishy, sour	sour/fermented	4.61	1.52	0.22	1.16	0.42	0.30	4.81	0.20	0.22	0.33
29	2-Methylpyridine	1236	PDD	sweaty, sour	sour/fermented	0.17	0.15	-	0.09	0.05	-	0.13	-	-	0.04
35	3-Methylpyridine	1309	PDD	green, earthy, nutty	green/vegetative	-	0.13	-	0.10	0.10	0.06	-	-	0.09	0.05
37	4-Methylpyridine	1317	PDD	green	green/vegetative	-	0.08	-	0.07	0.07	-	0.09	-	-	-
45	3-Ethylpyridine	1390	PDD	tobacco, oakmoss	roasted	0.26	0.26	0.14	0.26	0.22	0.26	0.27	0.17	0.37	0.21
59	3-Vinylpyridine	1488	PDD	-	-	-	-	-	0.23	0.13	-	-	-	-	-
79	2-Acetylpyridine	1613	PDD	popcorn, tobacco	roasted	0.34	0.44	0.62	0.55	0.53	0.67	0.41	0.73	0.75	0.56
84	2-Acetyl-6-methylpyridine	1649	PDD	roasted, coffee, cocoa	roasted	-	-	0.24	0.20	0.17	0.25	-	0.30	-	-
95	*N*-Acetyl-4(H)-pyridine	1720	PDD	-	-	0.82	1.11	1.36	1.24	1.20	1.51	1.07	1.38	1.51	1.38
102	4-Acetylpyridine	1805	PDD	burnt, coffee	roasted	-	-	0.33	-	-	0.37	-	0.39	-	-
	$\sum^{\;}\mathrm{Pyridine}\;\mathrm{and}\;\mathrm{derivatives}$					**6.29**	**3.73**	**2.91**	**3.94**	**2.89**	**3.42**	**6.84**	**3.17**	**2.94**	**2.57**
**Carbonic Acids**															
52	Acetic acid	1458	MA	acidic, pungent, cheesy	sour/fermented	1.20	0.88	0.19	0.58	0.26	0.31	0.99	0.25	1.00	0.40
69	Propanoic acid	1544	MA	acidic, pungent, cheesy	sour/fermented	0.24	-	-	-	-	-	-	-	-	-
83	4-Hydroxy-butanoic acid	1648	HCA	-	-	0.33	0.25	0.37	0.42	0.44	0.52	0.59	0.32	0.78	0.38
90	3-Methyl-butanoic acid	1675	MA	cheesy, sour, sweaty	sour/fermented	0.98	0.55	-	0.24	-	-	0.47	-	0.21	-
101	2-Butenoic acid (Crotonic acid)	1765	MA	milky	sour/fermented	0.21	-	-	-	-	-	-	-	-	-
103	3-Methyl-2-butenoic acid	1823	MA	phenolic, dairy, green	other	0.71	0.79	0.66	0.86	0.80	0.93	1.10	0.52	-	0.82
108	Hexanoic acid	1858	FA	sour, fatty, sweaty	sour/fermented	-	0.10	-	0.08	-	-	-	-	0.04	0.07
126	Octanoic Acid	2073	FA	sour, fatty, sweaty	sour/fermented	-	0.07	0.14	-	-	-	-	-	-	-
128	Nonanoic acid	2182	FA	sour, fatty, sweaty	sour/fermented	0.07	0.07	0.12	-	0.14	0.26	0.05	0.14	0.08	0.04
130	Decanoic acid	2288	FA	unpleasant rancid sour fatty citrus	sour/fermented	-	0.03	0.08	-	0.07	0.12	0.04	0.10	0.03	-
132	Dodecanoic acid	2510	FA	fatty, waxy	sour/fermented	-	0.02	-	-	-	-	-	-	-	-
	$\sum^{\;}\mathrm{Monocarbonic}\;\mathrm{acids}$					**3.74**	**2.76**	**1.56**	**2.18**	**1.71**	**2.14**	**3.24**	**1.33**	**2.14**	**1.71**
**Aldehydes and Ketones**															
2	Acetaldehyde	720	AA	fruity, fresh, aldehydic	fruity	0.13	0.02	-	0.05	-	-	-	-	-	-
4	2-Methylpropanal	817	AA	aldehydic, fresh, floral, pungent	other	0.52	-	-	-	-	-	-	-	-	-
7	2-Butanone	906	AO	acetone, ethereal	other	0.22	-	-	-	-	-	-	-	-	-
8	2-Methylbutanal	920	AA	musty, cocoa, coffee, nutty	other	0.59	0.15	-	0.07	0.01	-	0.07	-	-	0.04
9	3-Methylbutanal	924	AA	aldehydic, fruity, green	fruity	0.56	0.13	-	0.07	0.02	-	0.04	-	-	0.03
11	2,3-Butanedione	983	αDK	sweet, caramel, buttery	sweet	0.39	0.02	-	0.02	-	-	0.02	-	-	0.02
12	3-Pentanone	986	AO	ethereal, acetone	other	0.10	-	-	-	-	-	-	-	-	-
15	(*Z*)-2-Butenal	1041	AE	pungent, suffocating	other	0.12	-	-	0.04	-	-	-	-	-	-
16	3-Hexanone	1067	AO	fruity, sweet	fruity	0.05	-	-	-	-	-	-	-	-	-
17	2,3-Pentanedione	1072	αDK	sweet, caramel, buttery	sweet	0.96	0.13	0.07	0.10	0.04	0.05	0.39	0.10	-	0.09
19	Hexanal	1093	AA	green, fresh, fatty	green/vegetative	0.04	0.04	*-*	0.02	0.01	-	0.03	-	-	0.01
20	(E)-2-Methyl-2-butenal,	1103	AE	green, fresh, fruity	green/vegetative	0.14	0.03	*-*	-	-	-	0.06	-	-	-
21	2,3-Hexanedione	1137	αHK	buttery, sweet, caramel	sweet	0.19	0.05	*-*	0.03	-	-	0.09	-	-	0.02
22	3,4-Hexanedione	1146	αDK	buttery, sweet, caramel	sweet	0.12	0.04	*-*	0.01	-	-	0.05	-	-	0.01
34	3-Hydroxy-2-butanone (acetoin)	1293	αHK	buttery, creamy, dairy	sour/fermented	0.46	0.11	-	0.08	-	-	0.17	-	-	-
36	1-Hydroxy-2-propanone	1312	αHK	caramel, sweet	sweet	0.74	-	-	-	-	-	0.27	-	-	-
44	1-Hydroxy-2-butanone	1386	αHK	coffee, sweet	sweet	0.20	0.04	-	-	-	-	0.14	-	-	-
66	Benzaldehyde	1528	AAD	fruity, sweet, bitter almond	fruity	0.39	0.39	0.48	0.46	0.47	0.52	0.40	0.55	0.57	0.45
75	3,6-Heptanedione	1577	γDK	-	-	0.22	0.22	0.34	0.28	0.27	0.26	0.22	0.33	0.38	0.35
85	Benzeneacetaldehyde	1658	AD	honey, sweet, floral, green	sweet	*-*	-	0.26	-	-	-	-	-	-	-
86	2,4-Nonadienal, (E,E)-	1661	AKD	fatty, green	green/vegetative	-	-	-	-	-	-	-	1.16	-	-
97	2-Cyclohexene-1,4-dione	1760	CD	-	-	0.23	0.22	0.39	0.31	0.32	0.38	0.28	0.41	0.39	0.32
106	trans-*β*-Damascenone	1830	NK	floral, sweet, fruity	floral	-	-	0.20	0.14	-	0.14	0.12	0.13	-	-
116	*α*-Εthylidene-benzeneacetaldehyde	1934	AD	musty, sweet, cocoa	other	-	-	0.07	0.08	-	-	0.06	0.09	-	-
	$\sum^{\;}\mathrm{Aldehydes}\;\mathrm{and}\;\mathrm{ketones}$					**6.37**	**1.59**	**1.81**	**1.76**	**1.14**	**1.35**	**2.41**	**2.77**	**1.34**	**1.34**
**Phenol and Derivatives**															
110	2-Methoxy-phenol (guaiacol)	1867	PHD	smoky, burnt, spicy	roasted	0.78	0.83	1.45	1.26	1.08	1.15	0.84	1.58	1.32	1.18
121	Phenol	2010	PH	phenolic, plastic, rubbery	other	0.62	0.55	1.04	0.89	0.84	0.94	0.72	1.07	0.82	0.83
123	4-Ethyl-2-methoxy-phenol (4-ethylguaiacol)	2036	PHD	spicy, phenolic	spices	0.14	0.12	0.22	0.23	0.16	0.29	0.18	0.25	0.20	0.22
129	2-Methoxy-4-vinylphenol (4-Vinylguaiacol)	2202	PHD	spicy, clove, phenolic	spices	0.48	0.38	1.41	0.84	0.71	1.25	0.70	1.22	0.52	0.92
134	Vanillin	2591	PHD	sweet, vanilla	sweet	0.01	0.01	0.04	0.02	0.03	0.04	0.02	0.07	0.03	0.03
	$\sum^{\;}\mathrm{Phenol}\;\mathrm{and}\;\mathrm{derivatives}$					**2.03**	**1.89**	**4.16**	**3.24**	**2.82**	**3.67**	**2.46**	**4.19**	**2.89**	**3.18**
**Cyclopentene and Derivatives**															
42	2-Cyclopenten-1-one	1368	CP	-	-	0.18	-	-	-	-	-	0.16	-	-	-
43	2-Methyl-2-cyclopenten-1-one	1383	CPD	-	-	0.13	0.11	-	0.08	0.06	-	0.10	-	-	0.05
58	3,4,5-Trimethyl-2-cyclopenten-1-one	1483	CPD	-	-	0.16	0.17	-	0.09	0.08	0.08	-	-	0.12	0.10
76	2-Cyclopentene-1,4-dione	1583	CPD	-	-	0.33	0.00	0.67	-	0.62	-	-	0.55	-	-
99	2-Hydroxy-2-cyclopenten-1-one	1771	CPD	maple, caramel	sweet	0.35	0.35	-	0.40	-	-	-	-	-	-
113	3-Ethyl-2-hydroxy-2-cyclopenten-1-one	1897	CPD	sweet, caramel, maple	sweet	0.35	0.37	0.53	0.51	0.48	0.52	0.40	0.57	0.56	0.48
	$\sum^{\;}\mathrm{Cyclopentene}\;\mathrm{and}\;\mathrm{derivatives}$					**1.50**	**1.00**	**1.20**	**1.08**	**1.24**	**0.60**	**0.66**	**1.12**	**0.68**	**0.63**
**Pyranone Derivatives**															
118	Maltol	1972	PND	sweet, caramel, cotton candy	sweet	1.69	1.82	2.91	2.79	2.54	3.49	2.11	3.76	3.42	2.74
	$\sum^{\;}\mathrm{Pyranone}\;\mathrm{derivatives}$					**1.69**	**1.82**	**2.91**	**2.79**	**2.54**	**3.49**	**2.11**	**3.76**	**3.42**	**2.74**
**Dihydrofuranone Derivatives**															
32	Dihydro-2-methyl-3(2H)-Furanone	1277	DFD	nutty, bready, sweet	nutty/cocoa	0.72	0.18	-	0.12	-	0.05	0.54	-	0.08	0.06
87	2,5-Dihydro-3,5-dimethyl-2-furanone	1665	DFD	-	-	0.36	0.41	-	0.38	0.50	-	0.31	-	0.41	0.47
124	2,5-Dimethyl-4-hydroxy-3(2*H*)-furanone (furaneol)	2041	DFD	caramel, sweet, cotton candy	sweet	0.30	0.34	0.65	0.55	0.49	0.56	0.35	0.53	0.38	0.47
125	5-Acetyldihydro-2(3H)-furanone	2068	DFD	winey	sour/fermented	0.21	0.18	-	0.30	0.33	0.38	0.29	0.00	0.28	0.33
	$\sum^{\;}\mathrm{Dihydrofuranone}\;\mathrm{derivatives}$					**1.59**	**1.11**	**0.65**	**1.35**	**1.32**	**0.99**	**1.49**	**0.53**	**1.15**	**1.33**
**Dialkylsufides and Disulfides**															
60	2-(Methylthio)methylfuran (Furfuryl methyl sulfide)	1496	DS	alliaceous, vegetable	green/vegetative	0.40	0.43	0.16	0.31	0.27	0.28	0.27	0.20	0.37	0.30
104	2-[(Methyldithio)methyl]furan (Furfuryl methyl disulfide)	1825	DS	sulfurous, coffee, roasted	roasted	-	0.23	0.19	0.24	0.22	0.26	0.22	0.25	0.29	0.23
	$\sum^{\;}\mathrm{Diakylsulfides}\;\mathrm{and}\;-\mathrm{disulfides}$					**0.40**	**0.66**	**0.35**	**0.55**	**0.49**	**0.54**	**0.49**	**0.45**	**0.66**	**0.53**
**Thiols**															
1	Methanethiol	699	TH	alliaceous, egg, cabbage, garlic	other	0.07	0.04	-	0.04	0.01	0.01	0.03	-	-	0.04
50	2-Furfurylthiol	1440	TH	roasted, fresh coffee	roasted	0.31	0.32	0.16	0.32	0.15	0.25	0.16	0.15	0.21	0.18
	$\sum^{\;}\mathrm{Thiols}\;\mathrm{and}\;\mathrm{mercaptans}$					**0.38**	**0.36**	**0.16**	**0.36**	**0.16**	**0.26**	**0.19**	**0.15**	**0.21**	**0.22**
**Thiophene and Derivatives**															
13	Thiophene	1031	TPH	sulfurus, alliaceous, garlic	other	0.02	-	-	-	-	-	-	-	-	-
100	3-Acetylthiophene	1773	TPHD	-	-	-	-	0.27	-	-	0.35	0.00	0.28	0.32	0.32
105	3-Acetyl-2,5-dimethylthiophene	1827	TPHD	burnt, roasted	roasted	-	0.10	-	0.22	0.18	0.00	0.00	0.00	0.20	0.20
117	2-Thiophenemethanol	1947	TPHD	savory, coffee, roasted	roasted	0.23	0.19	0.25	0.26	0.23	0.29	0.23	0.24	0.19	0.24
	$\sum^{\;}\mathrm{Thiophenes}\;\mathrm{and}\;\mathrm{derivatives}$					**0.25**	**0.29**	**0.52**	**0.48**	**0.41**	**0.64**	**0.23**	**0.52**	**0.71**	**0.76**
**Alcohols**															
112	Benzyl alcohol	1878	ALC	floral	floral	-	-	-	0.10	0.08	-	0.24	-	0.10	0.09
115	Phenylethyl Alcohol	1916	ALC	floral, rose	floral	0.22	0.25	0.37	0.42	0.41	0.45	0.36	-	0.33	0.40
	$\sum^{\;}\mathrm{Alcohols}$					**0.22**	**0.25**	**0.37**	**0.52**	**0.49**	**0.45**	**0.60**	**-**	**0.43**	**0.49**
**Monoterpenes**															
24	*β*-Myrcene	1175	MT	spicy, peppery, terpenic	spices	0.07	0.07	0.19	0.05	0.05	0.05	0.08	0.20	-	0.07
26	*d*-Limonene	1210	MT	citrus, orange, fresh	fruity	0.23	0.20	0.28	0.16	0.10	0.08	0.23	0.29	0.08	0.14
70	Linalool	1555	MT	floral, sweet, citrus	floral	-	-	0.16	-	-	-	0.14	0.16	-	-
	$\sum^{\;}\mathrm{Monoterpenes}$					**0.30**	**0.27**	**0.63**	**0.21**	**0.15**	**0.13**	**0.45**	**0.65**	**0.08**	**0.21**
***γ*-Lactones**															
83	*γ*-Butyrolactone	1646	γLC	creamy, fatty, caramel	sweet	0.31	0.25	0.12	0.15	0.21	0.19	0.26	0.29	0.42	0,17
98	2(3H)-Furanone (*γ*-Crotonolactone)	1762	DHF	buttery	sour/fermented	0.28	0.27	-	-	-	-	-	-	-	-
127	2-Hydroxy-*γ*-butyrolactone	2180	γLC	-	-	0.05	0.05	-	0.06	0.05	-	-	-	-	0.04
	$\sum^{\;}\gamma -\mathrm{Lactones}$					**0.64**	**0.57**	**0.12**	**0.21**	**0.26**	**0.19**	**0.26**	**0.29**	**0.42**	**0.21**

^a^ Volatile compounds are presented in order of elution on the DB-Wax column. ^b^ Retention index. ^c^ AA: alkanal; AAD: aromatic aldehyde; AD: aldehyde; *α*DK: *α*-diketone; AE: alkenal; *α*HK: *α*-hydroxyketone; AKD: alkadienal; ALC: alcohol; AO: alkenone; CD: cyclohexanone derivative; CP: cyclopentanone; CPD: cyclopentanone derivative; DFD: dihydrofuranone derivative; DHF: dihydrofuranone; DS: diakylsulfide; *γ*DK: *γ*-diketone; ES: ester; F: furan; FA: fatty acid; FD: furan derivative; FND: furanone derivative; HCA: hydroxycarbonic acid; *γ*LC: γ-lactone MA; monocarbonic acid; MT: monoterpene; MTL: monoterpenol; NK: norisoprenoid ketone; PD: pyridine; PDD: pyridine derivative; PH: phenol; PHD: phenol derivative; PND: pyranone derivative; PR: pyrrole; PRD: pyrrole derivative; PZ: pyrazine; PZD: pyrazine derivative; TH: thiol; TPH: thiophene; TPHD: thiophene derivative; ^d^ Odor descriptions are from Good Scents Company Information System [23], Flavornet [24], and The Pherobase [25] online databases. ^e^ Expressed as relative percentage of each compound peak area to the total GC-MS peak area (*n*=3) ^f^ **-**: not found.

## Supplementary Materials

The following are available online, Figure S1: Typical time–temperature roasting curve.

## Sample Availability

Not available.
