# Supplementary material for: Single Origin Coffee Aroma: From Optimized Flavor Protocols and Coffee Customization to Instrumental Volatile Characterization and Chemometrics
Source: Molecules. 2021 Jul 29;26(15):4609. doi: 10.3390/molecules26154609 (PMC8346979; doi:10.3390/molecules26154609)
Supplement: Supplementary file 1 [file molecules-26-04609-s001.zip › molecules-1304200-supplementary.pdf]

Single Origin Coffee Aroma: From Optimized Flavor Protocols and Coffee Customization to Instrumental Volatile Characterization and Chemometrics

Panagiota Zakidou [1], Fotini Plati [1], Anthia Matsakidou [1], Evdoxia-Maria Varka [2], Georgios Blekas [1] and Adamantini Paraskevopoulou [1,*]

[1] *Laboratory of Food Chemistry and Technology, School of Chemistry, Aristotle University of Thessaloniki, 54124 Thessaloniki, Greece*
[2] *AVEK S.A., Naupliou 10-14, Metamorphosi, 14452 Athens, Greece*

* Correspondence: adparask@chem.auth.gr

Supplemmentary Material

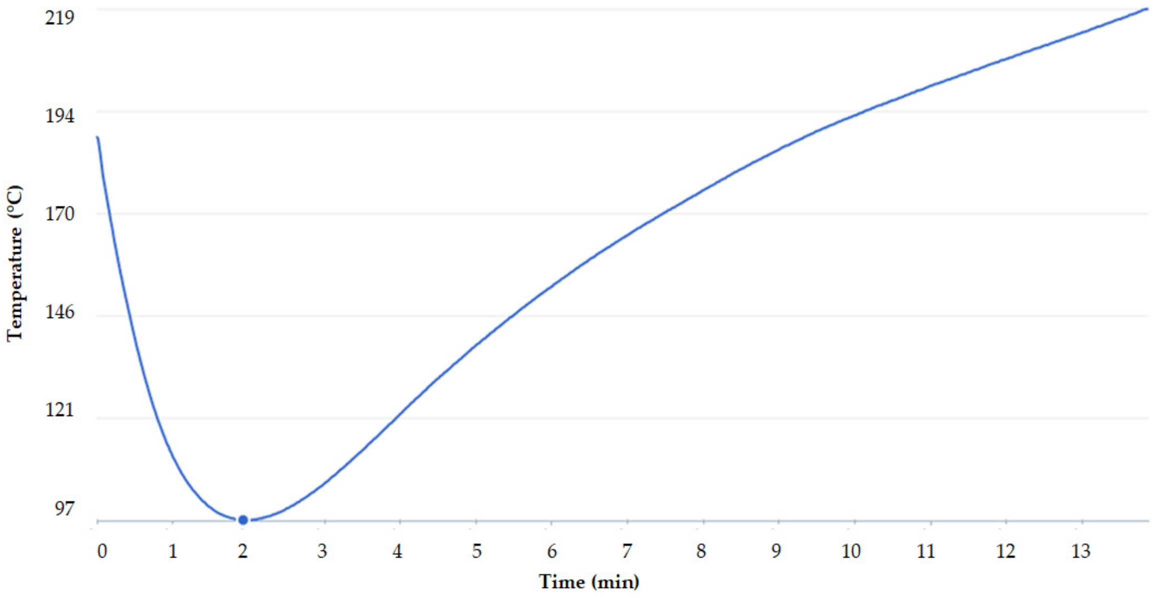

Figure S1: Typical time–temperature roasting curve
